# Veronal: A New Hypnotic
1Alfred C. Jordan, M.D. Brit. Med. Jour., March 5, 1904.


**Published:** 1904-07-16

**Authors:** 


### VERONAL: A NEW HYPNOTIC.[1]

Veronal is a colourless crystalline substance, sparingly soluble in cold water, readily soluble in hot water. It is most conveniently given in tablets, which can be dissolved in hot milk. The doses range from 4 to 40 grains, but in most cases a dose of $7\frac{1}{2}$ grains is sufficient to produce sleep within an hour, and lasting some seven or eight hours with, perhaps, an occasional interruption. The smaller doses were found to act admirably in weakly women suffering from advanced phthisis or from heart disease. When there is actual physical discomfort 15 grains may be needed to produce sleep, and in cases with severe pain, such, for example, as acute rheumatism or sciatica, veronal, like most of its fellows, cannot be successfully substituted for morphine. In the active mental excitement of delirium tremens, general paralysis, or epilepsy as much as 50 grains have been ordered with good effect, the patients not only being promptly calmed, but continuing in a more or less sleepy condition the whole of the following day. In some few instances a slight antipyrin-like rash has been observed, but with this exception veronal appears to be free from unpleasant after-effects. It has no action on the great vital centres, nor does it disturb the blood-pressure. It appears, also, that it does not create any "want" in the system, as when taken repeatedly the dose has rather to be diminished than increased, and there seems to be little or no tendency to the establishment of a veronal habit.

[1] Alfred C. Jordan, M.D. Brit. Med. Jour., March 5, 1904.

